# Recall Rates of Total Knee Arthroplasty Devices Are Dependent on the FDA Approval Process

**DOI:** 10.7759/cureus.9744

**Published:** 2020-08-14

**Authors:** Carl Pellerin, Micah Adamson, Cory Janney

**Affiliations:** 1 Department of Orthopaedic Surgery and Rehabilitation, University of Texas Medical Branch, Galveston, USA; 2 Department Orthopaedic Surgery, University of California Los Angeles Medical Center, Santa Monica, USA; 3 Department of Orthopaedic Surgery and Rehabilitation, Naval Medical Center San Diego, San Diego, USA

**Keywords:** device recall, fda, knee arthroplasty, orthopedic surgery

## Abstract

Introduction

The medical device industry has grown substantially in recent years. There is limited research examining orthopedic subspecialties and the recall of orthopedic devices. We hypothesize that knee arthroplasty devices cleared through the Food and Drug Administration (FDA) 510(k)-notification process would have a higher recall rate than the premarket approval (PMA) process.

Methods

The FDA database was thoroughly queried for all knee arthroplasty surgical devices from January 1, 2007 through December 31, 2017. Recalled devices were analyzed by manufacturer, type of implant, recall class, manufacturer-determined reason, FDA-determined reason, quantity affected, submission type, and distribution within the United States or internationally.

Results

Out of over 30,000 medical devices on the market, a total of 300 knee arthroplasty devices from 18 different companies were recalled during the time frame of this study. Tibial components accounted for 35.33% of devices, polyethylene implants for 38.67%, and femoral components for 15%. The most common reason for recall was device design (n = 134, 44.67%), followed by process control (n = 32, 10.67%). Of the 300 knee arthroplasty devices recalled, 267 (89.0%) were cleared through the 510(k) premarket notification process and 33 (11.0%) devices were approved through the PMA process.

Conclusions

A larger proportion of knee arthroplasty surgical devices cleared through the 510(k) process were recalled compared to implants approved through the stricter PMA process. Changing the 510(k) process may enable manufacturers to improve upon the safety of their devices.

## Introduction

The Food and Drug Administration (FDA) defines a recall as a method of removing or correcting products that violate rules and regulations administered by the FDA. By recalling faulty medical devices, distributors and manufacturers fulfil their duties to protect public health and well-being from products that present health risks, deceive the public, or are, in fact, defective [1,2]. Manufacturers typically perform recalls voluntarily as guided by 21 CFR part 7. However, 21 CFR part 810, the Medical Device Recall Authority, grants the FDA authority to issue a recall order when importers and manufacturers fail to withdraw a device that is considered a risk to public safety.

Medical devices may be approved through the premarket approval (PMA) process or cleared through the 510(k) premarket notification process. A PMA application requires scientific and regulatory evidence that establishes the safety and efficacy of a device for its intended use(s). This process can be costly and time consuming, as it requires sufficient scientific evidence for authorization [3]. FDA protocols then allow up to 180 days to review the submitted product for approval; however, this process generally takes longer [4]. In contrast, the 510(k) premarket notification is an expedited process that generally exempts medical devices from clinical trial requirements. To be cleared through the 510(k) process, new products must be deemed “substantially equivalent” to predicate devices that have already entered the market [5]. To be considered substantially equivalent, a device must perform in a manner similar to that of predicate devices in its intended use, technological characteristics, safety, and effectiveness. Between 2000 and 2009, over 30,000 medical devices were cleared through the 510(k) premarket notification process, compared to a little over 300 devices approved through the PMA process [6].

The medical device industry has grown substantially in recent years, increasing from $85 million in 2001 to $146 billion in 2009 in the United States alone [7]. While a portion of this growth may be attributed to the increased use of medical devices, a large part was driven by new competitors entering the market [6]. The influence of different types of medical devices on the United States’ economy varies significantly [8]. In the field of orthopedics, the majority of medical devices are used for procedures such as joint replacement, with a projected U.S. market of $10.3 billion in 2018, and fracture management, with a projected U.S. market of $4.3 billion in 2015 [3,9,10].

The number of recalls has also increased as the medical device industry continues to grow; 20,093 recalls were administered for 1,641 manufacturers between November 2002 and December 2012 [11]. Although there has been much debate over increasing the market’s regulations, recalls have not declined in recent years. In fact, the FDA processed an additional 13,937 medical device recalls between 2013 and 2017 [12]. Furthermore, the first quarter of 2018 experienced a 126% increase in device recalls, citing software issues in 23% of all recalls during this short time [13].

Orthopedic devices account for 12% of all medical device recalls in the United States [14]. From 2007 through 2017, 161 foot and ankle medical devices were recalled. Of these, 158 (98.1%) had been cleared through the 510(k) process, one (0.62%) through the PMA process, and submission methods for two products were not listed [15]. Although orthopedics accounts for a large percentage of medical device recalls, there is limited research examining orthopedic subspecialties. No previous research was found in the literature that examined medical device recalls in regards to knee arthroplasty. As such, this paper aims to identify knee arthroplasty device recalls and evaluate the reasons behind their removal from the market. We hypothesize that knee arthroplasty devices cleared through the 510(k)-notification process would have a recall rate than the PMA process.

## Materials and methods

The total number of orthopedic devices approved through PMA or cleared through the 510(k) premarket notification was determined by searching the FDA database from January 1, 2007 to December 31, 2017 for medical devices approved by an “orthopedic panel”. The FDA database was queried for all knee arthroplasty surgical devices during this time frame [2]. Duplicates were removed. A list of devices specific to the knee was compiled and evaluated to identify knee arthroplasty implants. We defined “implant” as a permanent device that functions to reinforce or provide fixation of bone, or to replace the articulating surface of a joint. The implant had to be unique to knee arthroplasty; these included femoral components, polyethylene inserts, tibial trays, stems, augments, metaphyseal sleeves and cones, and screws necessary for assembling the above components. We excluded devices that did not meet the definition of an implant, nonarthroplasty-specific implants (e.g., surgical guides, suture anchors, and prosthetic limbs) unless used for assembling components, and implants recalled secondary to issues with packaging. The remaining recalls were analyzed by manufacturer, type of implant (e.g., femoral components, tibial components, polyethylene implants, inserts, sleeves, spacers, etc.), recall class, manufacturer-determined reason for recall, FDA-determined reason, quantity affected, submission type, and distribution within the United States or internationally.

## Results

A total of 984 medical devices specific to the knee were identified during the time period. A total of 300 (30.49%) implants that met the criteria for a knee arthroplasty implant were recalled from January 1, 2007 through December 31, 2017. There were 18 different companies who manufactured these recalled devices. The most common reason for recall was device design (Table 1), accounting for 134 of the 300 device recalls (44.67%). For instance, 68,384 NexGen Complete Knee Solution MIS Tibial Components were recalled under device design due to loosening of the implanted device, leading to possible revision surgery [16]. Moreover, the Zimmer Gender Solutions Natural-Knee Flex System was categorized under device design, and 26,404 products were removed from market secondary to difficulty inserting the polyethylene into the tibial baseplate, causing intraoperative damage to the inserted instrument [17].

**Table 1:** Categories of devices that were recalled from market from January 1, 2007 through December 31, 2017. PMA: premarket approval.

FDA Determined Reason	Quantity	Percentage
Device design	134	44.67%
Process control	32	10.67%
PMA	26	8.67%
Process design	23	7.67%
Nonconforming material/component	16	5.33%
Employee error	14	4.67%
Storage	14	4.67%
Use error	11	3.67%
Component design/selection	9	3.00%
Equipment maintenance	9	3.00%
Packaging process control	4	1.33%
Mix-up of materials/components	3	1.00%
Under investigation by firm	2	0.67%
Unknown/undetermined by firm	2	0.67%
Process change control	1	0.33%
Total	300	100.00%

The second-most common reason for recall was process control, accounting for 32 recalls (10.67%). PMA, process design, and nonconforming materials/components accounted for 26 (8.67%), 23 (7.67%), and 16 (5.33%) recalls, respectively (Figure 2). The Zimmer Natural-Knee II System Modular Cemented Tibial Baseplate was recalled due to the tibial polyethyelene not affixing to the baseplate as intended. Employee error made 14 recalls (4.67%), 4 of which were due to products being inadequately polished, increasing risk of wear and polyethylene debris generation [18]. Products recalled due to packing process control (1.33%), mix-up of materials/components (1.00%), and process change control (0.33%) accounted for the least amount of recalls. Two devices were still under investigation by the FDA [19,20].

**Figure 1:**
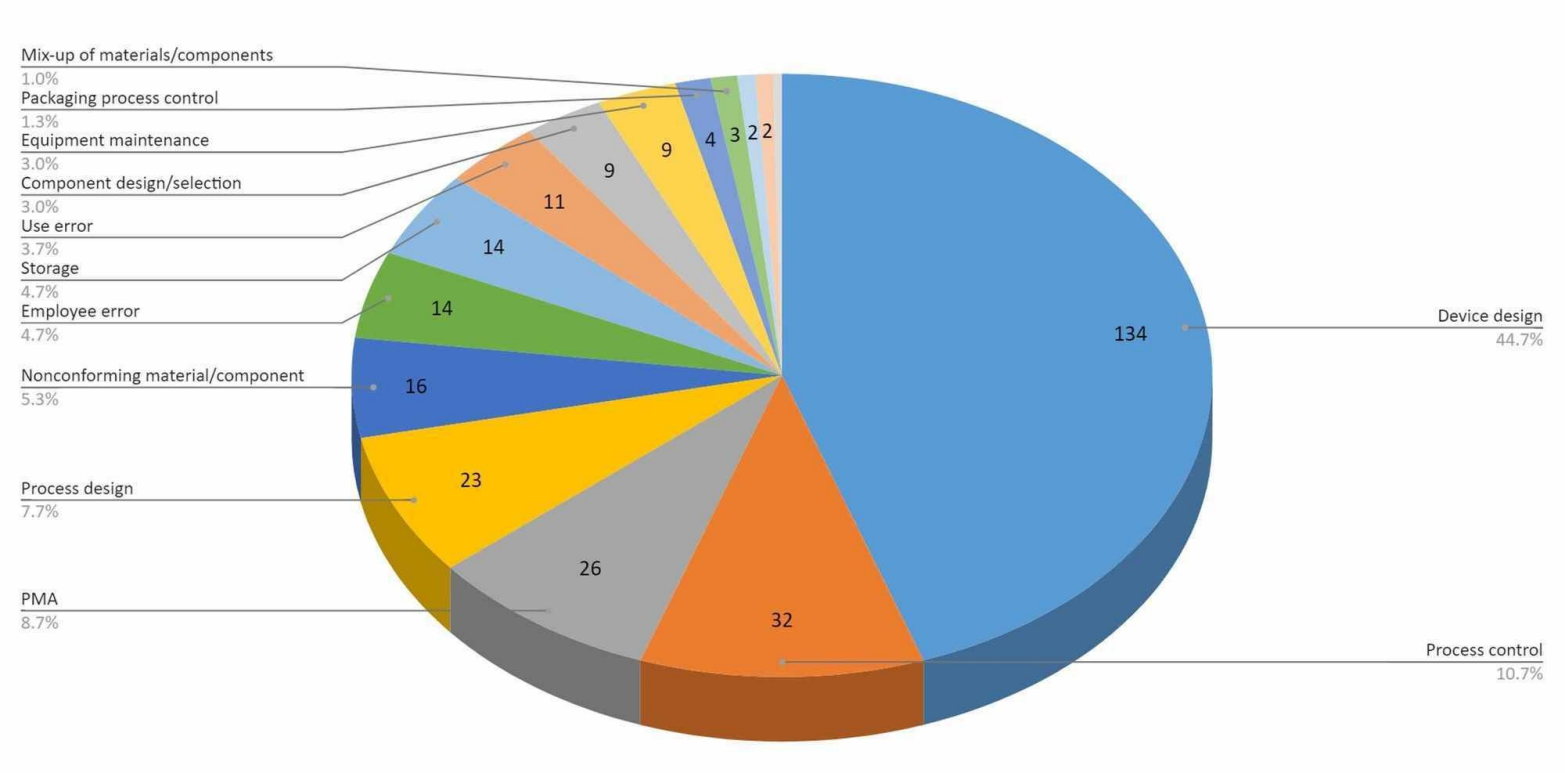
FDA-determined reasons for recalls of the devices from January 1, 2007 through December 31, 2017. PMA: premarket approval.

When evaluating the recalled devices based on manufacturer-determined reason, which may differ with the FDA-determined reason, 114 of the 300 products (38.0%) were recalled secondary to loosening or breaking (intraoperatively or postoperatively). The Natural-Knee II Durasul All-Poly Patella was recalled after 16 reports of shearing patellar pegs among the 109,386 distributed units (0.0137%), which the FDA categorized under component design/selection [21]. This device was responsible for the largest number of individual units being recalled out of all knee arthroplasty devices. Conversely, 10 different products tied for the least number of products being recalled, with only one item removed from market.

**Figure 2:**
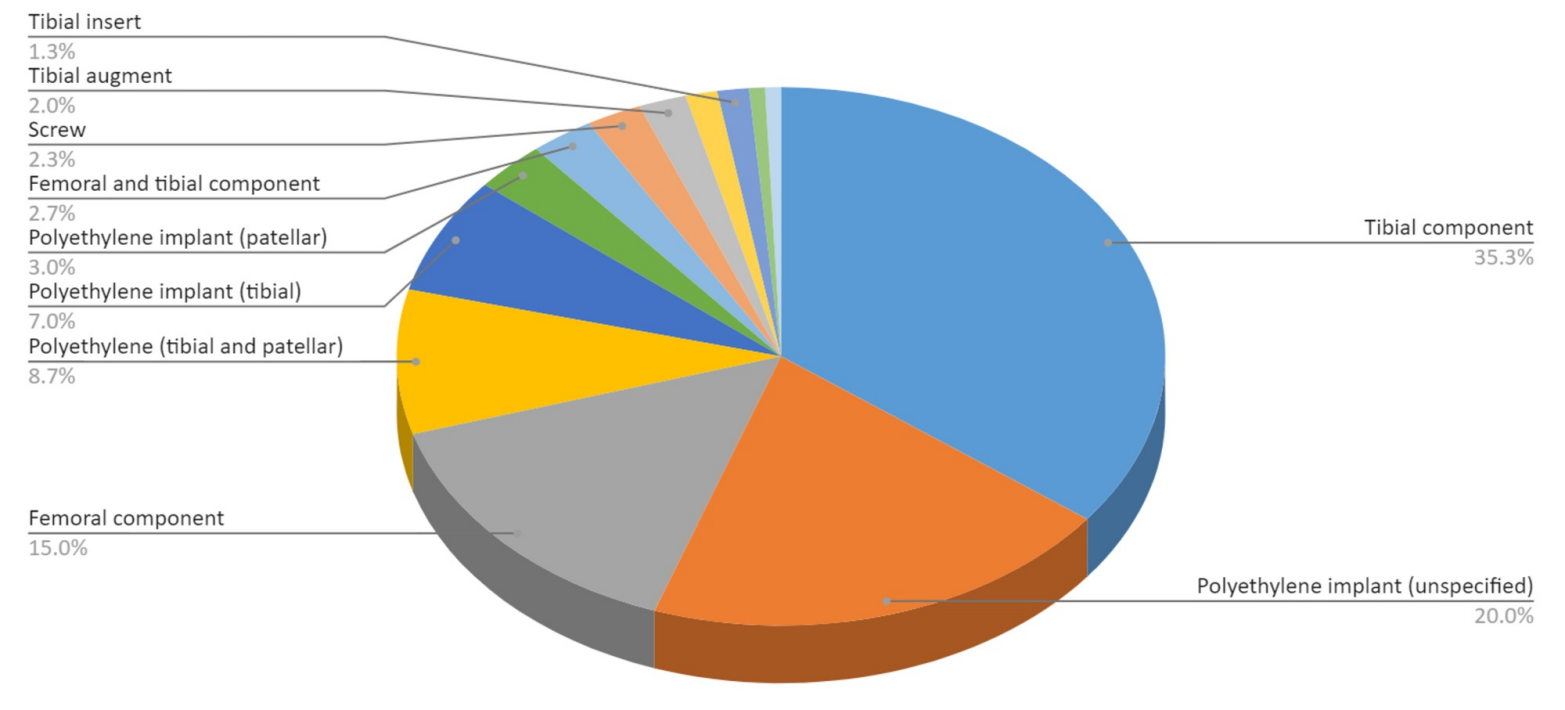
Categorization of recalled products by type of implant.

The recalled knee arthroplasty medical devices were separated by total quantity of each recalled implant (Table 2 and Figure 2). Tibial components made up 106 (35.33%) of the 300 recalled implants. Polyethylene implants accounted for 116 implants (38.67%): 21 tibial, 9 patellar, 26 both tibial and patellar together, and 60 unspecified. Femoral components accounted for 45 devices (15%). Implants with both femoral and tibial components combined accounted for eight devices (2.67%). Screws, tibial augments, diaphyseal sleeves, tibial inserts, tibial spacers, and an unicompartmental knee system accounted for the remaining 25 devices (8.33%).

**Table 2:** Categorization of recalled products by type of implant.

Categories of Recalled Implants	Quantity	Percentage
Tibial component	106	35.33%
Polyethylene implant (unspecified)	60	20.00%
Femoral component	45	15.00%
Polyethylene (tibial and patellar)	26	8.67%
Polyethylene implant (tibial)	21	7.00%
Polyethylene implant (patellar)	9	3.00%
Femoral and tibial component	8	2.67%
Screw	7	2.33%
Tibial augment	6	2.00%
Diaphysial sleeve	4	1.33%
Tibial insert	4	1.33%
Tibial spacer	2	0.67%
Unicompartmental knee system	2	0.67%
Total	300	100.00%

**Figure 3:**
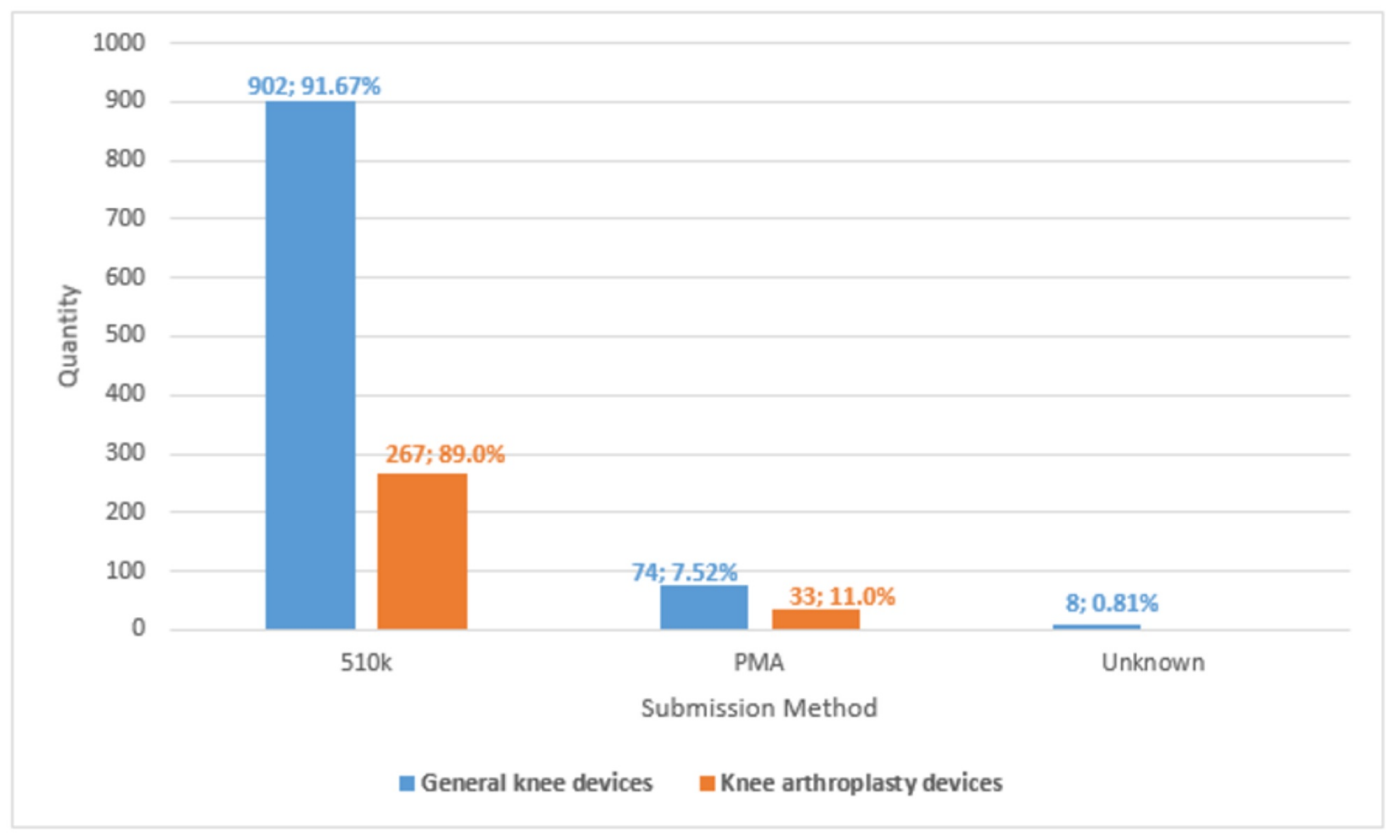
FDA approval process utilized by knee implants relative to general knee devices recalled during selected time frame. PMA: premarket approval.

Between January 1, 2007 and December 31, 2017, 6,758 devices orthopedic devices were approved: 5,833 (86.3%) via 510(k) premarket notification and 925 (13.7%) via PMA. Of the 300 knee arthroplasty devices recalled, 267 (89.00%) were cleared through the 510(k) premarket notification process. Conversely, 33 (11.00%) devices were approved through the PMA process (Figure 3). The average recall from posted date to termination date was about 448.83 days long.

## Discussion

Between January 1992 and December 2012, 77,164 medical devices were cleared through the 510(k) process, of which 8,345 (11%) were orthopedic devices. Conversely, 19,139 medical devices were approved through the PMA process, with 646 pertaining to the field of orthopedics. Within the same time period, the top 20 companies accountable for the majority of device recalls were responsible for 8,202 and 7,789 devices entering market through the 510(k) and PMA processes, respectively. From 2002 to 2012, these 20 companies also initiated 1,461 recalls for devices cleared via the 510(k) process and 121 recalls for devices approved via the PMA process. These recalls accounted for 17.8% of all 510(k)-cleared devices and 1.6% of PMA-cleared devices. Thus, orthopedic devices were recalled 11.5 times more often for 510(k)-cleared devices than PMA-approved devices. When evaluating by the individual medical device classes, the odds ratios were 3.5 for class I devices, 13.2 for class II devices, and 8.5 for class III devices [11].

Between 2015 and 2019, Day et al. found that orthopedic devices constituted a significant percentage of total medical device recalls, ranging from 11.8% to 21.5% [11]. In this time frame, the most common reasons for orthopedic recalls in general were packaging errors (33%), manufacturing errors (24%), and flawed device designs (24%). The least common reasons were misleading marketing and software issues. We discovered notable differences when comparing general orthopedic recalls with knee arthroplasty recalls. In order to provide good value-based care, choosing a total knee implant with a good track record is the responsibility of the surgeon [22].

Manufacture of medical devices and orthopedic implants are multibillion dollar industries [23,24]. The number of primary and revision total knee arthroplasties performed annually is expected to increase significantly during the next decade [25,26]. The process of bringing a new implant to market in the United States can be cumbersome [3]. Companies have a financial incentive to minimize the cost associated with this process. The 510(k) process allows for a product claimed to be “substantially equivalent” to become FDA-approved through a much simpler and faster process than the PMA process [5]. According to our data, between January 1, 2007 and December 31, 2017, 6,758 devices orthopedic devices were approved: 5,833 (86.3%) via 510(k) premarket notification and 925 (13.7%) via PMA. Of the knee arthroplasty devices recalled, 89% were cleared through the 510(k) premarket notification process, whereas 11% of the devices were authorized through the PMA process. Although our findings suggest that devices cleared through the 510(k) premarket notification process may have a slightly increased recall rate (86.3% approved vs. 89% recalled), it is not significant enough to claim that products cleared through 510(k) are more likely to be recalled. Regardless, the large number of devices recalled each year present significant health and safety concerns.

There has been a slight decline in the percentage of orthopedic products cleared through the 510(k) method. From 1992 to 2012, the number of orthopedic devices approved via this process decreased from 91% to 88% [11]. This relatively insignificant decrease is concerning. Zuckerman et al. determined that the less stringent 510(k) method contributed the largest percentage of devices recalled due to life-threatening hazards [27]. Thus, enacting more standardized and stringent reviews may reduce the percentage of recalls.

Class III medical devices need to be approved through the PMA process as they carry the greatest potential risks. However, many high-risk orthopedic devices have been miscategorized under class II, bypassing the PMA requirements [27]. Between 1992 and 2012, nearly 94% of orthopedic devices were categorized as class II [11]. Due to the risks that faulty surgical devices impose on public health and the difficulty of surgical revision, the criteria for class III devices should be more inclusive. Companies should be prevented from circumventing safety protocols. Moreover, once devices are approved by the FDA, manufacturers can make minor modifications without undergoing further safety analysis. Although these manufacturers need to submit a “supplement” to the previously approved submission application, incremental improvements may be made to a device as long as it goes through a short-term review. Consequently, devices may be modified dozens of times, and the relevance and value of the original clinical data become less reliable [28]. Thus, product modifications should be limited, unless companies are willing to undergo additional safety testing.

In November 2018, the FDA established quality improvements in the 510(k)-screening method in regards to safety and effectiveness. The updated guidelines included the following: (1) increasing premarket requirements for the submission process, (2) refusing to accept incomplete applications, (3) enacting more standardized and stringent reviews, (4) terminating the use of 510(k) for class III devices, and (5) decreasing the number of devices that may be used as 510(k) predicates [29,30]. With the recent modifications to the 510(k)-notification process, further research is necessary to evaluate how recent changes will impact device recalls and public health. 

This study has limitations. It focused only on knee arthroplasty devices identified through a search of the FDA database, and it is possible that some devices were not identified during the search process. Another limitation is that some recalled implants were limited to certain batch numbers. If the FDA classified a device recall as “other”, the device was placed in a category that best fit the manufacturer’s recall description. The FDA-determined reason for recall was listed as “PMA” for 26 devices, without any further explanation. Thus, “PMA” was included in the data. Two devices were still under investigation by the FDA. Moreover, the FDA categorized two DePuy orthopedic femoral implants as “unknown/undetermined”.

## Conclusions

Our findings suggest that most total knee-related implants are recalled due to issues of device design, process control, and PMA. Of the recalled knee arthroplasty devices, tibial components and polyethylene implants accounted for almost three-quarters of recalled devices. Based on manufacturer-determined reasons, more than one-third of medical devices presented the risk of breaking or loosening intraoperatively or postoperatively. With faulty medical devices putting patient safety at risk, it is concerning that most recalls take over a year to remove products from circulation.

A large proportion of recalled knee arthroplasty surgical devices cleared through the 510(k) process were recalled compared to the number of implants approved through the more stringent PMA process. Although our findings suggest that devices cleared through the 510(k) premarket notification process have a slightly increased recall rate, it is not significant enough to claim that products cleared through 510(k) are more likely to be recalled. Surgeons should stay abreast of recalls among the products that they use frequently, educate patients regarding any issues associated with the products, and closely monitor patient progress postoperatively. Changing the 510(k) process may enable manufacturers to improve upon the safety of their devices.
